# Case Report: Hepatic sinusoidal obstruction syndrome caused by *Gynura **s**egetum*

**DOI:** 10.3389/fmed.2026.1889644

**Published:** 2026-07-22

**Authors:** Bochen Xiang, Qiang Liu, Xiangcheng Dai, Xiaolin Zhou

**Affiliations:** 1 Department of Infectious Diseases, Yichang Central People’s Hospital, The First College of Clinical Medical Science, China Three Gorges University, Yichang, China; 2 Department of Infectious Diseases, Yichang Hospital, Union Hospital, Tongji Medical College, Huazhong University of Science and Technology, Yichang, China

**Keywords:** *Gynura segetum*, hepatic sinusoidal obstruction syndrome, liver injury, portal hypertension, pyrrolizidine alkaloids

## Abstract

Hepatic sinusoidal obstruction syndrome (HSOS) is a hepatic vascular disease initiated by injury to hepatic sinusoidal endothelial cells. Its main clinical manifestations include hepatomegaly, elevated bilirubin, ascites and weight gain. The incidence of HSOS has been increasing year by year. Nevertheless, due to its non-specific clinical manifestations, limited effective diagnostic methods and imperfect relevant diagnostic criteria, it is highly likely to be misdiagnosed as other liver diseases. This study reports a case of HSOS induced by ingestion of *Gynura segetum*.

## Introduction

HSOS is a vascular liver disease associated with poor prognosis. Due to the non-specific nature of its clinical manifestations and insufficient awareness among practitioners at primary healthcare institutions, misdiagnosis and missed diagnosis are common, leading to delayed optimal treatment (1). This report presents a case of severe HSOS caused by the ingestion of *Gynura segetum* (colloquially known as “tu sanqi”), a plant containing pyrrolizidine alkaloids (PAs). The patient was initially diagnosed with “decompensated cirrhosis” at an outside hospital. Upon admission to our institution, she presented with severe hepatic dysfunction; contrast-enhanced abdominal computed tomography (CT) revealed marked hepatomegaly with a typical “mottled” enhancement pattern, and the final diagnosis was established as HSOS. Despite aggressive treatment, the patient’s condition continued to deteriorate, and she died of liver failure within 2 weeks after discharge. Through clinical analysis and discussion of this case, we aim to raise clinical awareness of HSOS, strengthen early recognition of severe cases, and provide a reference for optimizing diagnostic and therapeutic strategies.

## Case presentation

**Figure 1:**
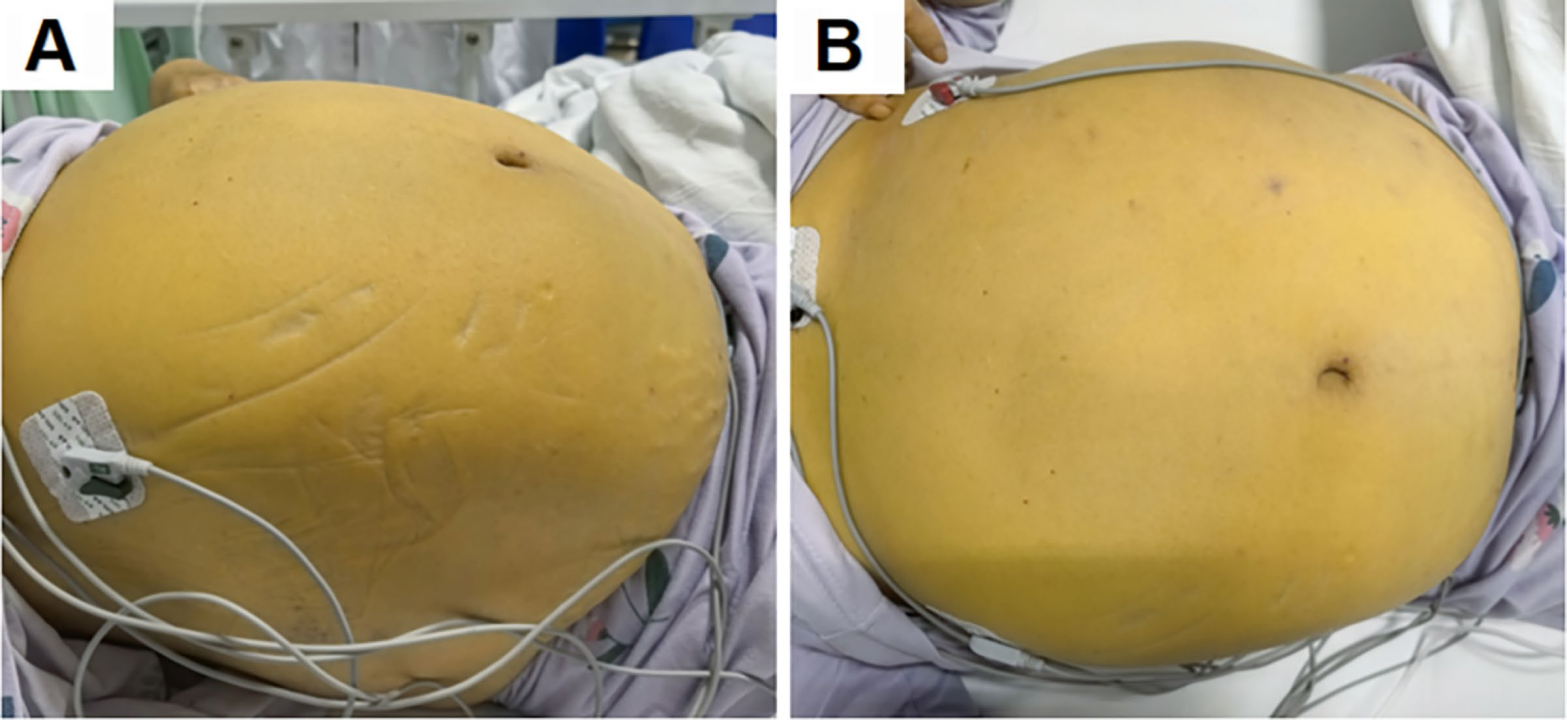
Lateral **(A)** and anterior **(B)** views of the patient’s abdomen.

A 69-year-old female ingested 3 g of “Tusanqi powder” three times daily for 6 months. Subsequently, the patient developed fatigue, anorexia, and abdominal distension (Figure 1).

The patient had begun intermittent ingestion of “tu sanqi” around March 2024. On admission, liver function tests and coagulation parameters were markedly abnormal (Table 1). Serological markers for hepatitis viruses were negative (hepatitis B surface antigen negative, anti-hepatitis C virus antibody negative). Epstein–Barr virus IgM antibody and cytomegalovirus IgM antibody were negative. Autoimmune liver disease-related antibodies — including anti-nuclear envelope glycoprotein antibody, anti-liver–kidney microsomal antibody type 1, anti-soluble liver antigen antibody, anti-soluble acidic nuclear protein antibody, anti-mitochondrial M2 antibody, and anti-liver cytosol antigen type 1 antibody — were all negative. Extractable nuclear antigen antibodies and anti-neutrophil cytoplasmic antibodies were within normal limits. Serum ceruloplasmin levels were unremarkable.

**Table 1:** Laboratory findings on admission.

Parameter	Result	Unit	Reference range
Alanine aminotransferase (ALT)	44↑	U/L	9–50
Aspartate aminotransferase (AST)	61↑	U/L	15–40
Total bilirubin (TBIL)	431.0↑	μmol/L	0–21.0
Direct bilirubin (DBIL)	223.4↑	μmol/L	0–6.8
Total protein (TP)	59.10↓	g/L	65–85
Albumin (ALB)	34.80↓	g/L	40–55
Total bile acid (TBA)	305.9↑	μmol/L	0–10
Alkaline phosphatase (ALP)	176↑	U/L	56–119
γ-Glutamyltransferase (GGT)	261↑	U/L	10–60
Prothrombin time (PT)	54.8↑	s	11–15
Prothrombin activity (PTA)	12.0↓	%	70–150
International normalized ratio (INR)	6.50↑		0.80–1.20
Activated partial thromboplastin time (APTT)	140.9↑	s	32.0–45.0
Fibrinogen (Fib)	<0.5↓	g/L	2.00–4.00
Thrombin time (TT)	45.1↑	s	14.0–21.0
Platelets	108↓	10^9^/L	125–350
MELD score	40		
Child-Pugh score	C		

ALT, alanine aminotransferase; AST, aspartate aminotransferase; TP, total protein; ALB, albumin; TBA, total bile acid; ALP, alkaline phosphatase; GGT, γ-glutamyltransferase; Fib, fibrinogen.

**Figure 2:**
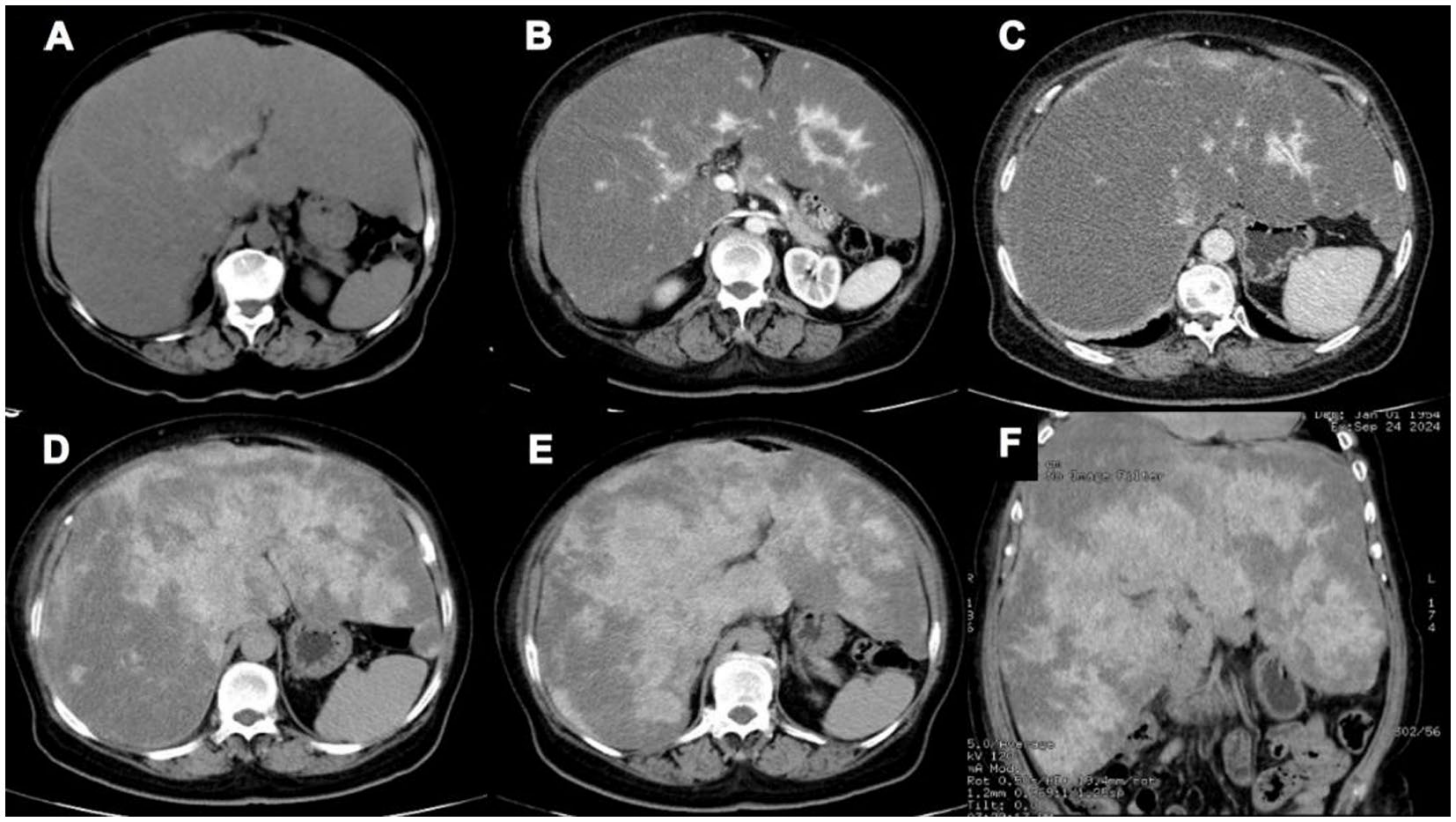
Plain and contrast-enhanced abdominal CT. Reduced hepatic parenchymal density with wavy hepatic margin **(A)**; heterogeneous enhancement of portions of the hepatic parenchyma **(B)**; slender intrahepatic segment of the inferior vena cava, attenuated left and middle hepatic veins, and indistinct right hepatic vein **(C)**; heterogeneous hepatic parenchymal density on delayed-phase imaging with a “geographic map–like” heterogeneous enhancement pattern **(D,E)**; markedly enlarged liver extending to the iliac fossa **(F)**.

Contrast-enhanced abdominal CT demonstrated the following findings (Figure 2): markedly enlarged liver with irregular contour; hepatic parenchymal density lower than that of the spleen on the same cross-section (CT value approximately 28 HU); disproportionate hepatic lobe ratios; wavy liver margin; widened hepatic fissures; a low-density nodule approximately 0.8 cm in longest diameter in the left hepatic lobe; heterogeneous enhancement of portions of the hepatic parenchyma on delayed-phase imaging, presenting a “geographic map–like” heterogeneous pattern; slender intrahepatic segment of the inferior vena cava; attenuated left and middle hepatic veins; indistinct right hepatic vein; normal portal vein diameter; non-enlarged gallbladder with focal wall thickening and a small amount of pericholecystic fluid; unremarkable size, morphology, and parenchymal density of the spleen, pancreas, and bilateral kidneys, with no abnormal enhancement; no evidence of retroperitoneal lymphadenopathy.

In this case, given the patient’s severely compromised coagulation function, the risk of percutaneous liver biopsy was deemed prohibitively high; after thorough communication with the patient and her family, the procedure was not performed. Although liver biopsy and pyrrole-protein adduct testing were unavailable/not feasible according to the “Nanjing Criteria” (2) (Table 2), after exclusion of other causative factors, “tu sanqi” was identified as the most probable causative agent.

**Table 2:** Diagnostic criteria for HSOS.

Diagnostic criteria	Contents
Baltimore criteria (17)	Within 21 days after hematopoietic stem cell transplantation, serum total bilirubin ≥ 34.2 μmol/L, accompanied by at least 2 of the following 3 manifestations: Ascites Hepatomegaly with right upper quadrant pain Weight gain exceeding 5% of baseline body weight
Modified seattle criteria (18)	Within 20 days after hematopoietic stem cell transplantation, presence of 2 of the following 3 manifestations: Hepatomegaly or right upper quadrant pain Serum total bilirubin ≥ 34.2 μmol/L Ascites, or weight gain exceeding 2% of baseline body weight due to fluid and sodium retention
Nanjing criteria (2)	Definite history of ingestion of pyrrolizidine alkaloid-containing plants; the diagnosis can be established by satisfying all 3 items below or confirmed by liver pathology, with liver injury induced by other known etiologies excluded: Abdominal distension and/or right upper quadrant pain, hepatomegaly and ascites Elevated serum total bilirubin or other abnormal liver function parameters Typical manifestations on contrast-enhanced CT or MRI

Aggressive treatment was initiated immediately upon admission, comprising hepatoprotective therapy (polyene phosphatidylcholine, intravenous infusion; ademetionine 1,4-butanedisulfonate, intravenous infusion), regulation of bile acid metabolism (ursodeoxycholic acid, oral), blood transfusion to improve coagulation function, and symptomatic supportive care. Given the patient’s severe coagulopathy and markedly elevated bilirubin levels, artificial liver support, bilirubin adsorption, and liver transplantation were considered as potential interventions to improve hepatic function. However, published evidence in post-hematopoietic stem cell transplantation SOS has documented that non-relapse mortality correlates closely with peak bilirubin levels and the presence of multi-organ failure, and that patients with a prothrombin activity as low as 12% may deteriorate fatally within days. In this context, coagulopathy itself serves both as a marker of disease severity and as a contraindication to invasive procedures. Given the patient’s profoundly impaired coagulation function and severe thrombocytopenia, the hemorrhagic risk associated with artificial liver support or any related invasive intervention was deemed prohibitive. Following thorough discussion with the patient and her family, a conservative management approach was adopted.

**Figure 3:**
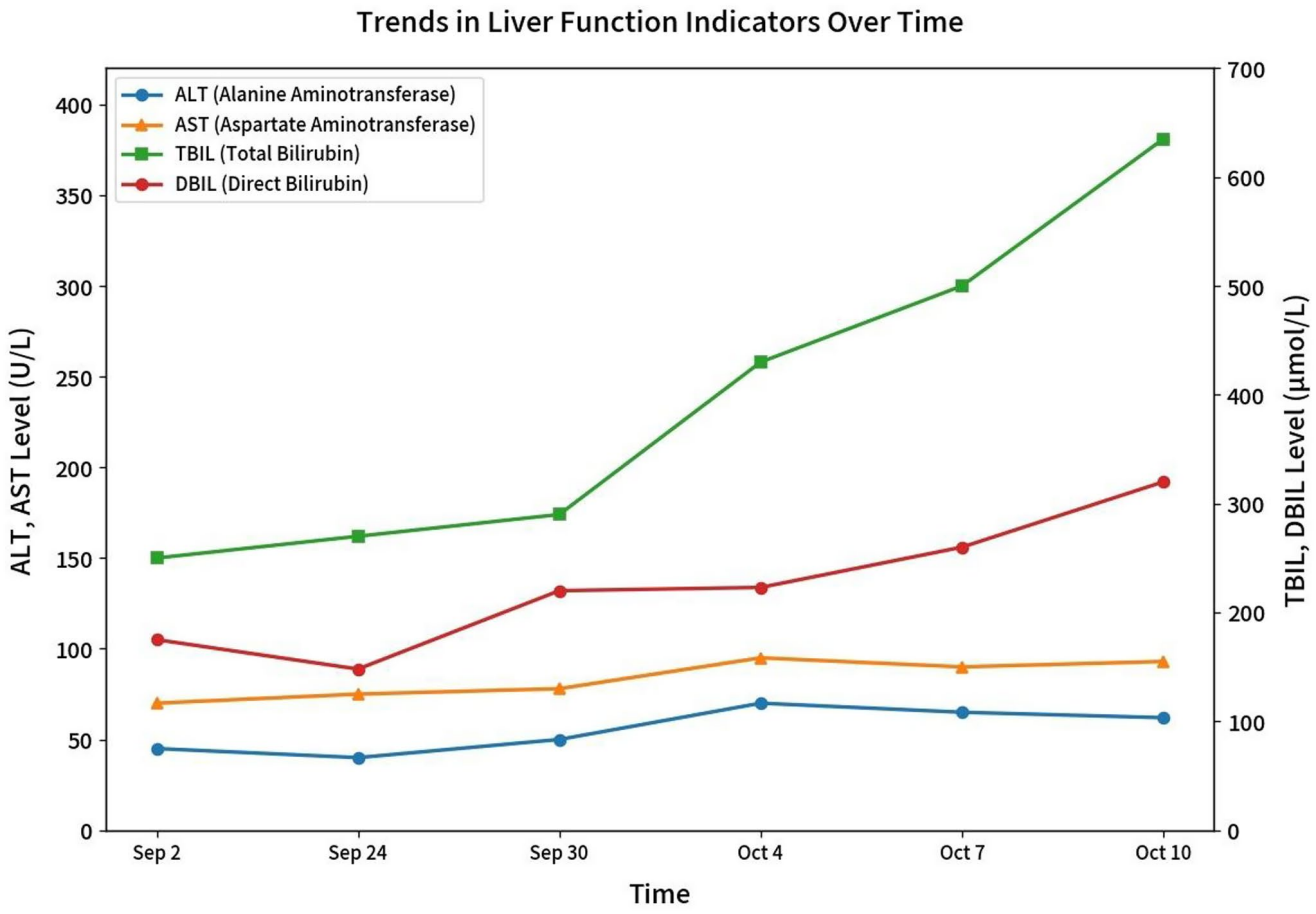
Line graph depicting changes in liver function parameters over the course of hospitalization.

Repeat liver function tests on day 9 of hospitalization showed: alanine aminotransferase (ALT) 39 U/L (↑), aspartate aminotransferase (AST) 66 U/L (↑), total bilirubin (TBIL) 632.7 μmol/L (↑), direct bilirubin (DBIL) 317 μmol/L (↑), total protein (TP) 59.1 g/L (↓), albumin (ALB) 34.8 g/L (↓), total bile acid (TBA) 302.5 μmol/L (↑), alkaline phosphatase (ALP) 200 U/L (↑), and *γ*-glutamyltransferase (GGT) 152 U/L. These findings revealed a striking bilirubin–enzyme dissociation pattern (Figure 3): despite only modest transaminase elevations (ALT 39 U/L, AST 66 U/L — near the upper limit of normal), total bilirubin had risen dramatically to 632.7 μmol/L, approximately 30-fold above the upper reference limit. This dissociation is pathognomonic of progressive hepatic sinusoidal obstruction with severe post-sinusoidal portal hypertension, where the predominant injury is to the sinusoidal endothelium and downstream hepatic outflow rather than to hepatocytes per se; the near-normal transaminases therefore do not reflect clinical stability but rather a shift in injury phenotype (3, 4). Simultaneously, fibrinogen remained critically depleted (<0.5 g/L) and prothrombin activity was profoundly suppressed (PTA 12%), indicating consumptive coagulopathy driven by ongoing endothelial activation and hepatic synthetic failure — a pattern consistent with a terminal coagulation–fibrinolysis dysregulation cascade (5). Collectively, these laboratory dynamics were indicative of fulminant hepatic insufficiency and an irreversible trajectory, reflecting an inadequate response to treatment. After renewed discussion of the clinical situation with the patient and her family, the patient opted to discontinue treatment and requested discharge.

Follow-up 2 weeks after discharge confirmed the patient had died.

## Discussion

Hepatic sinusoidal obstruction syndrome, formerly termed hepatic veno-occlusive disease (VOD), is characterized by concentric, non-thrombotic obliteration of the sinusoidal and central venous lumina, in the absence of primary or thrombotic hepatic vein disease (6). The condition typically presents with acute right upper quadrant pain, hepatomegaly, ascites, and rapid weight gain attributable to fluid retention. Diagnosis relies on a combination of medical history, clinical manifestations, laboratory findings, imaging studies, biomarkers, and hepatic histopathology (7). In developed countries, HSOS is predominantly caused by myeloablative conditioning prior to hematopoietic stem cell transplantation (HSCT) or oxaliplatin-based chemotherapy (8). In China, HSOS is mainly attributable to the ingestion of pyrrolizidine alkaloid-containing herbal medicines or dietary supplements (9).

In this case, the patient presented with nonspecific symptoms, including fatigue, poor appetite, and abdominal distension for more than 6 months, and was initially misdiagnosed with decompensated cirrhosis. Further evaluation revealed no history of alcohol use, hereditary disease, chronic portal hypertension, or autoimmune disorders. Serological tests for viral hepatitis and HIV were negative. Given the history of Tusanqi powder ingestion and characteristic imaging findings of hepatomegaly with patchy heterogeneous enhancement, all diagnostic criteria of the Nanjing Criteria were fulfilled, leading to a diagnosis of PA-induced HSOS (PA-HSOS). The most notable laboratory finding was the development of marked bilirubin–enzyme dissociation on hospital day 9. Because HSOS primarily damages sinusoidal endothelial cells rather than hepatocytes, progressive sinusoidal obstruction causes post-sinusoidal portal hypertension and impaired bile excretion, resulting in severe hyperbilirubinemia despite only mild aminotransferase elevation. Therefore, stable or declining aminotransferase levels accompanied by continuously rising bilirubin should not be interpreted as disease improvement but rather as a sign of advanced and potentially irreversible disease progression. The patient also developed severe coagulopathy, characterized by persistent fibrinogen depletion and declining prothrombin activity, reflecting ongoing endothelial injury, impaired hepatic synthesis of coagulation factors. This not only indicated a poor prognosis and high risk of fatal bleeding but also precluded anticoagulant therapy, one of the key treatments for PA-HSOS. Taken together, progressive bilirubin–enzyme dissociation and irreversible coagulation dysfunction are important indicators of end-stage PA-HSOS and impending liver failure. Recognition of these dynamic biochemical changes may facilitate earlier intervention and timely evaluation for liver transplantation before therapeutic opportunities are lost.

Regarding HSCT-associated HSOS, the incidence of sinusoidal obstruction syndrome (SOS) varies considerably across studies, largely owing to differences in patient characteristics, conditioning regimens, transplant center experience, and diagnostic criteria. The reported incidence is 3.1–8.7% following autologous HSCT and 8.9–14.0% following allogeneic HSCT; the pediatric population exhibits a higher incidence (10). The core pathophysiological mechanism involves chemo/radiotherapy-induced injury to hepatic sinusoidal endothelial cells, triggering coagulation–fibrinolysis dysregulation, hepatic sinusoidal microthrombosis, and post-sinusoidal portal hypertension — processes closely linked to complement overactivation (11). The 2023 European Bone Marrow Transplantation (EBMT) revised diagnostic criteria represent a key advance: they introduce a three-tier classification of suspected, clinical, and pathologically confirmed disease; integrate Doppler ultrasonography and hepatic elastography; enable early recognition of both classic-onset and late-onset cases; and stratify severity into mild, moderate, severe, and very severe grades, substantially improving diagnostic accuracy (12). Defibrotide remains the standard first-line treatment for severe SOS/VOD; early administration at adequate dosage significantly improves outcomes. Concurrent management of modifiable risk factors, optimization of supportive care, and individualized preventive and management strategies for special populations — including the elderly, patients undergoing second transplantation, and those receiving gene therapy — are also essential components (13).

In recent years, HSOS has gained broad recognition as a rare form of drug-induced liver injury (DILI). Despite extensive investigation in the HSCT setting, the applicability of that body of knowledge to drug-induced HSOS remains limited owing to etiological differences (14). PAs derived from *Gynura segetum* constitute the primary cause of herbal medicine-induced liver injury in China, capable of inducing severe HSOS with high mortality (15). PAs are metabolically activated by hepatic CYP450 enzymes to generate dehydrogenated products that form adducts with proteins and DNA, selectively injuring hepatic sinusoidal endothelial cells and thereby precipitating sinusoidal obstruction, hepatocyte apoptosis, and hepatic fibrosis; involvement of the immune, hematologic, and pulmonary systems has also been documented. Therapeutic strategies for PA-induced HSOS include strict fluid management, anticoagulation therapy, corticosteroids, transjugular intrahepatic portosystemic shunt (TIPS), and liver transplantation (3). A meta-analysis demonstrated that TIPS is a feasible and relatively safe treatment modality for HSOS, particularly in patients with PA-HSOS (16). In a recent separate investigation, a chronic PA-HSOS mouse model was established, from which *Serpine1* was identified as a potential therapeutic target; this finding suggests that future pharmacological interventions may be explored through the coagulation regulatory pathway (5).

Despite etiological heterogeneity, HSCT-associated and herbal medicine-induced HSOS converge on shared pathophysiological pathways, both characterized by hepatic sinusoidal endothelial injury, microvascular obstruction, and portal hypertension (12). The EBMT diagnostic criteria have substantially enhanced early recognition and severity stratification of the disease in the transplantation context; however, their applicability to toxin-induced HSOS remains limited. Developing a unified diagnostic and therapeutic framework that integrates endothelial injury biomarkers, exposure history, and dynamic clinical indicators may address this gap and advance the precise diagnosis and standardized management of HSOS across etiologies.

## Data Availability

The original contributions presented in the study are included in the article/supplementary material, further inquiries can be directed to the corresponding author.
